# Report of Cases of Cerebro Spinal Meningitis

**Published:** 1892-05

**Authors:** T. O. Hinebauch

**Affiliations:** Fargo, N. D.


### REPORT OF CASES OF CEREBRO SPINAL MENINGITIS.

By T. O. Hinebauch, Fargo, N. D.

On January 9th I was called, in consultation with Dr. Taylor, of Hillsboro, for the purpose of investigating a serious outbreak of a peculiar disease among a bunch of fifty-five work horses and mules belonging to a bonanza farmer. The following symptoms were noticed:

*History.*—The horses and mules appeared as well as usual. The first symptoms that directed the attendants' attention was the length of time it took the affected animals to drink, they standing at the watering trough for from a few minutes to half an hour, or even longer, apparently drinking all the time. On making an examination it was found that but little, if any, water was swallowed. After a variable time, from six or eight hours to two or three days, after the animals were first noticed to be ailing, they were down. During this period they would take feed, masticate it, and drop it into the manger again.

*Symptoms.*—Hanging of the head more or less, perspiration, profuse at times, and at others the coat would be nearly dry; listlessness, dull staggering gait; conjunctivæ slightly injected, mucous membranes somewhat reddened. Except during the process of mastication, a continual flow of clear, ropey saliva, occasionally, but rarely frothy; mouth fœtid in some cases, and sour odor. Urine normal, bowels somewhat constipated, feces hard, pellaty and dark colored. Ears and legs cold. Pulse weak, 40 to 48 per minute. Respiration somewhat increased. Temperature, 102 to 103 degrees, intestinal murmurs. Ausculation and percussion of the thoracic cavity showed a normal condition.

After a variable period of from one or two to three or four days the animal would fall down and be unable to rise again. In falling they reared up on their hind legs and fell over backwards; at first there was considerable uneasiness, but as they continued to lie upon the side the uneasiness gradually disappeared and they became comatose, only struggling at intervals, which varied more or less in duration. Their efforts gradually decreased, showing that the animal was gradually losing strength. This continued until death took place.

*Prognosis.*—In this particular outbreak all horses which became attacked died, there being seven in number. The best of medical treatment was adopted, but seemed to be of no avail. At the time that I was called in consultation, animals which had been exposed to the cause of the disease were given a good purgative, accompanied by full doses of nitrate of potash and carbolic acid, internally. Whether this treatment was beneficial or not is hard to tell, although after it was adopted not a single animal was attacked.

*Supposed Cause.*—On inquiring into the history of these cases nothing could be learned which seemed to bear upon the subject, the water being of the same nature as that which they had received for several years, coming from a well 150 to 200 feet deep, somewhat alkaline in nature, as more or less of the wells are in this part of the country. While looking through the barn we noticed a pit, and on making inquiry found it was for the purpose of storing carrots and other root crops. We also learned that two days previous to the first attack the pit had been emptied of frozen carrots, and it was also developed that the carrots were covered with a white mold, the tops being in a partially rotten condition, not having been removed when they were put into the pit. Of the animals attacked all had access to the carrots, which were thrown out into the yard, there being six or eight bushels thrown into a heap. A portion of the animals were not turned out, and did not have access to the carrots; not one of that number became affected. Taking the evidence into consideration, it seems that the whole trouble was undoubtedly due to the eating of musty or moldy carrots, which were partially frozen. The symptoms of the disease would also indicate that it is a brain difficulty. We would diagnose it as cerebro spinal meningitis.

*Post-mortem.*—The subject of the post-mortem was a bay mare, four years old, five the coming spring. When we first saw her she was lying on her right side, in a partially comatose condition, making feeble efforts to rise at intervals of fifteen or twenty minutes, although at that time she could not raise her head. She was destroyed by opening the carotid artery. The animal was left lying on its right side, the left hind leg removed, also the left front leg. The incision was then made along the lumbar region, down along the flank, and the first four false ribs were also removed, in that manner exposing the contents of the abdominal cavity. In cutting through the abdominal muscles, several small worms (oxyuris Vermicularis) were observed apparently embodied in the muscular tissue. As soon as the intestinal tract was exposed, numerous worms of the same variety were also observed lying on the outside of the large floating colon and small intestines. The general appearance of the intestines departed from the normal in that small, dark-colored spots, varying in size from a pin's head to a common white bean. These were dark, had a congested appearance, but did not yet show an inflammatory action. The stomach was full and distended. On making an incision, the contents resembled bile, had a greenish cast, and also an odor peculiar to bile. The contents of the intestines, with regard to consistency, normal. The last two feet of the small intestine contained considerable clay, which existed in small round balls about the size of a pea or bean, besides other portions, which were practically loose and floury or meal like. The liver, in appearance, normal. The same can also be said of the pancreas and spleen. On opening the intestinal tract, great quantities of worms of the variety described were noticed ; indeed, they were so numerous that they could scarcely fail to attract attention. I removed two inches of the intestines, took it to my laboratory, and had one of the students remove the worms. He removed and counted 500, and said, to all appearances, that they were as numerous as when he began, he having only selected the larger ones. The whole of the intestinal tract apparently was infected by these worms, no part being ex-exempt. Computing the number on that basis, we can readily see that they amounted to thousands, and it is a wonder that the horse could live, when we take into consideration that they had passed out of the tract, entering the abdominal cavity and muscles. The contents of the chest were normal, so far as could be observed. On exposing the spinal cord we found that it was inflamed from the base of the brain for four inches along its length. From there on congestion existed for about five or six inches more; there was an enormous amount of fluid surrounding the cord in the region of the medulla oblongata, which completely filled the space and undoubtedly must have been the result of exudation. On making a cross section of the cord just below the medulla oblongata, it was seen that the whole contents of the cord were highly congested and inflammation present in a greater or less degree.

